# 'The smoking toolkit study': a national study of smoking and smoking cessation in England

**DOI:** 10.1186/1471-2458-11-479

**Published:** 2011-06-18

**Authors:** Jennifer A Fidler, Lion Shahab, Oliver West, Martin J Jarvis, Andy McEwen, John A Stapleton, Eleni Vangeli, Robert West

**Affiliations:** 1 Cancer Research UK Health Behaviour Research Centre, Department of Epidemiology & Public Health, University College London, London, UK; 2 Tobacco Dependence Research Unit, Barts and The London School of Medicine and Dentistry, Queen Mary University of London, London, UK

## Abstract

**Background:**

Up-to-date data tracking of national smoking patterns and cessation-related behaviour is required to evaluate and inform tobacco control strategies. The Smoking Toolkit Study (STS) was designed for this role. This paper describes the methodology of the STS and examines as far as possible the representativeness of the samples.

**Methods:**

The STS consists of monthly, cross sectional household interviews of adults aged 16 and over in England with smokers and recent ex-smokers in each monthly wave followed up by postal questionnaires three and six months later. Between November 2006 and December 2010 the baseline survey was completed by 90,568 participants. STS demographic, prevalence and cigarette consumption estimates are compared with those from the Health Survey for England (HSE) and the General Lifestyle Survey (GLF) for 2007-2009.

**Results:**

Smoking prevalence estimates of all the surveys were similar from 2008 onwards (e.g 2008 STS = 22.0%, 95% C.I. = 21.4% to 22.6%, HSE = 21.7%, 95% C.I. = 20.9% to 22.6%, GLF = 20.8%, 95% C.I. = 19.7% to 21.9%), although there was heterogeneity in 2007 (chi-square = 50.30, p < 0.001). Some differences were observed across surveys within sociodemographic sub-groups, although largely in 2007. Cigarette consumption was virtually identical in all surveys and years.

**Conclusion:**

There is reason to believe that the STS findings (see http://www.smokinginengland.info) are generalisable to the adult population of England.

## Background

The UK is one of the leading countries with regard to policies aimed at reducing mortality and morbidity from tobacco smoking [1]. Up-to-date and detailed surveillance data are important for evaluating the effectiveness of the UK's tobacco control strategy, making recommendations concerning different methods of encouraging smoking cessation, and helping shape government and health service policy. Findings from the UK can also be used to inform policy internationally.

Several large-scale surveys collect data on tobacco control parameters (Table 1). These surveys all provide useful data, but have some limitations. First, most provide data which are out of date by up to two years at time of publication [2]. This limits their ability to inform tobacco control in a timely manner. Second, because the two principal surveys in England, the General Lifestyle Survey (GLF, formally the General Household Survey) and the Health Survey for England (HSE) cover a wide range of health and lifestyle topics, their data on smoking patterns and cessation-related activities are limited. By contrast, surveys such as the International Tobacco Control (ITC) [3] and ATTEMPT [4] cohort studies collect more smoking-related information. However, the UK samples are relatively small. In addition, the ATTEMPT study was limited to smokers interested in quitting between certain ages and is not ongoing. The ITC study follows a cohort of smokers annually but is somewhat limited due to the exclusion of recent ex-smokers from subsequent surveys and does not allow fine grain tracking. Thus while these studies are crucial to answering certain questions in the field of tobacco control, it was judged that there was a need for an additional study to provide ongoing, up-to-date national statistics tracking key parameters relating to tobacco smoking, especially prevalence, cessation, motivation to stop, and harm reduction, and to provide contextual information to help understand these data.

**Table 1:** Characteristics of tobacco control surveys

Survey	Sampling	Frequency of data collection	Tobacco control parameters measured
GHS/GLF	Nationally representative household surveys across the UK. N = ~ 14,500 adult smokers and non-smokers.	Annual, smoking data collected since 1974. From 2005, respondents followed-up for 4 years, with ~25% replaced each year.	Smoking prevalence; cigarette consumption; cigarette type; cigarette dependence; tar yield; age started smoking; desire to quit; demographics.

HSE	Nationally representative household surveys across England. N = 5000-15000 adults smokers and non-smokers.	Annual, since 1991. No follow-up.	Smoking prevalence; cigarette consumption; cigarette type; cigarette dependence; salivary cotinine (biochemical indicator of cigarette smoke intake); focus in 2007 on Smokefree legislation.

ONS	Nationally representative household surveys across the UK. N = ~1,800 adult smokers and non-smokers.	Monthly, since 1990. Basic smoking questions asked routinely in 2 months each year. Additional smoking questions included when requested.	Varies month-to-month and year-to-year. *At least yearly*: smoking prevalence, dependence, behaviour, and habits. *Previously requested*: attitudes towards smoking, quitting, and smoking restrictions; awareness of health-risks; attempts to quit; demographics.

ITC	Random telephone dialling across 20 countries (including UK and USA); telephone surveys. N = ~2,000 adult smokers per country.	Annual, since 2002. Yearly follow-up with replenishment for drop-outs and those who stop smoking.	Smoking behaviours and dependence; quitting behaviours; use of alternative nicotine products; attitudes towards and effects of label warnings, advertising, and taxation; health beliefs; demographics; other potential moderators.

ATTEMPT	Internet recruitment and assessment across the UK, USA, France, and Canada. N = ~2,000 adult smokers intending to quit in next 3 months.	Survey lasted 2.5 years. Tri-monthly follow-up.	Smoking behaviours and dependence; number and method of quit attempts; reasons for quitting; short-term health effects; weight-related measures; demographics.

GHS/GLF, General Lifestyle Survey (formerly General Household Survey); HSE, Health Survey for England; ONS, Office of National Statistics Opinions Survey (formerly Omnibus Survey); ITC, International Tobacco Control Policy Evaluation Project.

The Smoking Toolkit Study (STS) was designed to achieve this goal. Detailed data are collected on a wide range of smoking-related parameters at monthly intervals. The study is ongoing and has thus far produced a number of papers [5-17]. Up-to-date findings are also published on a dedicated website: http://www.smokinginengland.info. The purpose of this paper is twofold: 1) to provide a description of the STS methodology in greater detail than can be given in other papers, and 2) to establish the generalisability of the samples by comparing STS estimates with equivalent estimates from the two principle established national surveys, the GLF and the HSE, on demographics and two key tobacco control parameters, smoking prevalence and cigarette consumption.

## Method

The STS involves monthly cross-sectional household computer-assisted interviews, conducted by the British Market Research Bureau as part of their monthly omnibus survey, of approximately 1,800 adults aged 16 and over in England. Cigarette smokers and recent ex-smokers (who have smoked in the past year) who agree to be re-contacted are followed up three- and six-months later by postal questionnaire. Baseline data were first collected in November 2006, followed by three and six month postal follow-ups in February and May 2007, respectively. Due to funding constraints the three month postal questionnaire was discontinued in January 2010.

The baseline surveys use a form of random location sampling. England is split into 165,665 Output Areas, each comprising approximately 300 households. These Output Areas are stratified by ACORN characteristics (an established geo-demographic analysis of the population; http://www.caci.co.uk/acorn/ and geographic region then randomly selected to be included in an interviewer's list. Interviewers travel to the selected areas and perform computer assisted interviews with one participant aged over 16 per household until quotas based upon factors influencing the probability of being at home (working status, age, and gender) are fulfilled. Morning interviews are avoided to maximise participant availability. Random location sampling is considered superior to conventional quota sampling because the choice of properties approached is reduced by the random allocation of small output areas. However, interviewers can still choose which houses within these areas are most likely to fulfil their quotas, rather than being sent to specific households in advance. Response rates are not therefore appropriate to record, unlike random probability sampling, where interviewers have no choice as to the properties sampled and so response at each address can be recorded. The analysis uses the rim (marginal) weighting technique, an iterative sequence of weighting adjustments whereby separate nationally representative target profiles are set (for gender, working status, prevalence of children in the household, age, social grade and region) and the process repeated until all variables match the specified targets.

Smokers and recent ex-smokers are asked at baseline whether they are willing to be re-contacted. They are mailed a short follow-up questionnaire three months later, followed by a second at 6 months if they respond to the first. Half of the respondents at three months are randomly selected (after stratification by age and social grade) to be sent a postal saliva sample kit to complete and return with the questionnaire. The saliva is assayed for cotinine, the primary metabolite of nicotine, enabling biochemical assessment of smoking during the preceding few days [18]. Follow-up participants are given up to £5 remuneration (amounts varied slightly across the four years of the study) and one reminder letter is sent.

The study was conceived by researchers at the Cancer Research UK's Health Behaviour Research Centre, University College London, who continue to manage it. Ethical approval was granted by the University College London ethics committee.

### Samples

Between November 2006 and December 2010 a total of 90,568 participants completed the baseline survey (monthly range = 1,634-2,642). Of these 23,326 reported smoking within the last year. Response rates are not available for the baseline survey due to the sampling strategy used. Response rates are collected for the follow-ups. Of the total 15,536 eligible for follow-up at times when the 3-month questionnaire was scheduled, 14,025 were willing to be re-contacted (90%). Of these 4,755 (34%) returned a completed postal questionnaire. Of the 7,013 asked to provide a saliva sample 1,702 (24%) returned a questionnaire at three months. Sixty nine percent (n = 3,302) of those responding at three months also returned a six month questionnaire.

### Measures

A core set of key performance indicators are included in each STS survey (see Table 2 for assessments routinely included each month). Specific questions are added to the survey to address particular issues (e.g. to assess the impact of Smokefree legislation and public support for a levy on tobacco products to fund tobacco control initiatives). The postal follow-up questionnaire is much shorter. Questions include current smoking status, number of cigarettes smoked, attempts to stop and characteristics of those attempts, attitudes towards smoking, cutting down smoking behaviour and tobacco dependence.

**Table 2:** Key assessments of the Smoking Toolkit Study

Smoking status	Daily; non-daily; quit within the last year; quit more than a year ago; never smoked for a year or more; use of non-cigarette tobacco
Amount smoked and nicotine intake	Cigarettes or other tobacco products used per day, week, or month; salivary cotinine (follow-up only)

Nicotine dependence	Fagerstrom Test for Nicotine Dependence, strength of urge to smoke

Route to quit	Motivation to quit; triggers of quit attempts; barriers to quitting; attempts to quit; methods of quitting (including pharmacological and behavioural aids, planning in advance, pre-quit cutting down); success at quitting

Motivation to smoke	Attitudes, beliefs, and motives associated with smoking

Harm reduction	Prevalence of attempts to cut down but not quit; use of nicotine replacement therapy when cutting down and/or prohibited from smoking

Demographics	Gender; age, socio-economic status; geographic region

Smoking status and cigarettes smoked per day are analysed in the current paper. Smoking status was assessed with the following question: 'Which of the following best applies to you? I smoke cigarettes (including hand-rolled) every day, I smoke cigarettes (including hand-rolled), but not every day; I do not smoke cigarettes at all, but I do smoke tobacco of some kind (e.g. pipe or cigar); I have stopped smoking completely in the last year; I stopped smoking completely more than a year ago; I have never been a smoker (i.e. smoked for a year or more); Don't Know'. Those who responded that they smoked cigarettes every day or that they smoked cigarettes but not every day are coded as current cigarette smokers. Cigarette consumption is measured using the following question 'How many cigarettes per day do/did you usually smoke'. Those who do not smoke every day can give a figure per week or per month.

Socio-demographic information includes: gender, age, and social grade based on information about the occupation of the chief income earner, as used in the British National Readership Survey [19]. The social grade categories are: AB = higher and intermediate professional/managerial, C1 = supervisory, clerical, junior managerial/administrative/professional, C2 = skilled manual workers, D = semi-skilled and unskilled manual workers, and E = on state benefit, unemployed, lowest grade workers. These are dichotomised into ABC1 and C2DE in the current analyses.

### Statistical Analysis

We examined the heterogeneity of demographic characteristics, smoking prevalence and cigarette consumption estimates between the STS, GLF (English data only) and HSE using the meta-analysis program Comprehensive Meta-Analysis (CMA)[20] and the chi-square Q-test. Where there was evidence of differences, we used pair-wise chi-square or t-tests to examine these. No correction is made for multiple pair-wise comparisons.

HSE and GLF use the question 'Do you smoke cigarettes at all nowadays?' (Yes/No) to assess smoking status. From this question, national rates of cigarette smoking prevalence are estimated, overall and by gender, age and socioeconomic status. The mean number of cigarettes smoked per day was assessed in both the HSE and GLF surveys with the question 'About how many cigarettes a day do you usually smoke at weekends/on weekdays'. All data were weighted. Data from HSE and GLF were adjusted for clustering using complex samples procedures.

Although the STS has results for 2007-2010, full data are only available from the GLF and HSE to 2008. The 2009 HSE and GLF prevalence results are available in report format only [21,22]. For this analysis, 2009 percentages were taken from these published reports and the standard errors (confidence intervals) have been estimated from sample sizes and standard errors published for previous years.

Full details of the methodology for HSE and GLF are available in published reports [23-26] and online [27,28]. However, in brief, both surveys provide a sample representative of the population living in private households, using a multi-stage stratified probability design. Specific addresses are selected from chosen postcode sectors and letters sent to these households prior to an interviewer visit. All members of the household are then interviewed on a range of topics by trained interviewers using computer assisted interviewing.

## Results

Table 3 shows the demographic characteristics of baseline STS respondents in 2007 and 2008, when GLF and HSE data were also available. As might be expected with weighted data, there was no evidence of heterogeneity in the demographic characteristics of the three surveys in 2007, except for Socio-Economic Status (SES). Pair-wise comparisons showed that the GLF SES profile was significantly different from that of the STS (X2 = 47.7, p < 0.001) and the HSE (X2 = 7.9, p = 0.005). In 2008 all three surveys differed slightly in terms of SES, and there was also a lower proportion of men in GLF compared with both STS (X2 = 6.7, p = 0.010) and HSE (X2 = 11.3, p = 0.001).

**Table 3:** Demographic characteristics of the STS, HSE and GLF (percent, 95% confidence interval unless stated)

	2007	2008
**Male (%, 95% C.I.)**		
STS	48.5 (47.9 to 49.2)	48.5 (47.8 to 49.3)
GLF	48.6 (47.7 to 49.5)	47.1(46.4 to 47.8)
HSE	48.8 (47.9 to 49.7)	48.8 (48.2 to 49.5)
Heterogeneity	X^2 ^= 0.27, p = 0.875	X^2 ^= 12.7, p = 0.002
**Age (mean, 95% C.I.)**		
STS	46.4 (46.1 to 46.7)	46.3 (46.0 to 46.6)
GLF	46.4 (46.1 to 46.7)	47.3 (46.7 to 47.9)
HSE	46.4 (45.8 to 47.0)	46.3 (45.8 to 46.8)
Heterogeneity	X^2 ^= 0.00, p = 1.00	X^2 ^= 5.8, p = 0.056
Lower social grade (%, 95% C.I.)*		
STS	44.6 (43.9 to 45.2)	44.5 (43.8 to 45.3)
GLF	40.3 (39.4 to 41.3)	39.4 (38.0 to 40.8)
HSE	43.0 (41.4 to 44.6)	42.8 (41.7 to 43.9)
Heterogeneity	X^2 ^= 47.7, p < 0.001	X^2 ^= 38.9, p < 0.001

*STS = social grade, HSE = NSSEC (household) split Professional/Managerial/Intermediate & Routine/Manual

**Figure 1:**
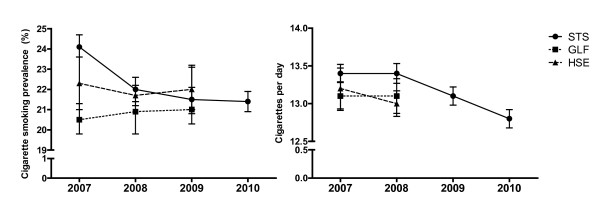
**Smoking prevalence and cigarettes per day by survey and year**.

Figure 1 shows the pattern of smoking prevalence and cigarette consumption across years for each study, including STS data up to 2010. The overall prevalence estimates differed between all three surveys in 2007, but not in other years (Table 4). STS prevalence was higher than for the HSE (X2 = 6.1, p = 0.014) and the GLF (X2 = 22.1, p < 0.001), and GLF prevalence was lower than the STS (above) and HSE (X2 = 5.3, p = 0.02). Differences were apparent for both genders. In 2007 there was also evidence of prevalence differences between the surveys for all age groups except those aged 65 plus. There was no consistent pattern to these differences. In 2008 there was only evidence of a difference between the surveys for 25-44 year olds, with the GLF giving a lower estimate than both STS (X2 = 4.1, p = 0.043) and HSE (X2 = 7.9, p = 0.005). For both 2007 and 2008 there were differences between the three surveys in prevalence estimates by socioeconomic status. Prevalence was higher in STS compared with GLF in 2007 in both higher SES (X2 = 12.5, p < 0.001) and lower SES (X2 = 25.6, p < 0.001) groups. In 2008 there was no difference between STS and GLF, but the STS estimate was lower than the HSE estimate in the higher SES group (X2 = 11.4, p = 0.001) and higher than the HSE estimate in the lower SES group (X2 = 7.1, p = 0.008).

**Table 4:** Cigarette smoking prevalence (percent, 95% CI) by sociodemographics for each survey from 2007-2009 where available

		2007	2008	2009†
**Total**	STS	24.1 (23.6 to 24.7)	22.0 (21.4 to 22.6)	21.5 (21.0 to 22.1)
**population**	GLF	20.5 (19.8 to 21.3)	20.9 (19.9 to 22.0)	21 (20.0 to 22.0)
	HSE	22.3 (21.0 to 23.6)	21.7 (20.9 to 22.6)	22 (20.4 to 23.6)
	Heterogeneity	X^2 ^= 50.3, p < 0.001	X^2 ^= 2.97, p = 0.227	X^2 ^= 1.3, p = 0.535
**Gender**				
Men	STS	25.6 (24.7 to 26.4)	22.9 (22.0 to 23.8)	22.6 (21.8 to 23.5)
	GLF	22.0 (20.8 to 23.2)	21.9 (20.6 to 23.3)	22 (20.7 to 23.3)
	HSE	23.8 (22.1 to 25.5)	23.7 (22.5 to 25.0)	24 (21.8 to 26.2)
	Heterogeneity	X^2 ^= 22.4, p < 0.001	X^2 ^= 3.7, p = 0.159	X^2 ^= 2.4, p = 0.306
Women	STS	22.8 (22.0 to 23.6)	21.2 (20.4 to 22.0)	20.5 (19.7 to 21.3)
	GLF	19.2 (18.2 to 20.3)	20.1 (18.9 to 21.3)	20 (18.8 to 21.2)
	HSE	20.8 (19.4 to 22.4)	19.9 (18.9 to 20.8)	20 (18.1 to 21.9)
	Heterogeneity	X^2 ^= 29.2, p < 0.001	X^2 ^= 4.7, p = 0.095	X^2 ^= 0.58, p = 0.750
**Age**				
16-24	STS	32.7 (31.2 to 34.3)	29.3 (27.7 to 31.0)	28.3 (26.8 to 29.9)
	GLF	27.2 (24.3 to 30.0)	27.6 (24.4 to 31.1)	-
	HSE	25.3 (21.8 to 29.2)	26.2 (23.9 to 28.6)	-
	Heterogeneity	X^2 ^= 20.2, < 0.001	X^2 ^= 4.6, p = 0.099	-
25-44	STS	28.2 (27.2 to 29.2)	27.1 (26.0 to 28.1)	26.3 (25.3 to 27.3)
	GLF	24.4 (23.0 to 25.8)	24.9 (23.1 to 26.8)	-
	HSE	27.9 (25.7 to 30.0)	28.4 (26.8 to 30.0)	-
	Heterogeneity	X^2 ^= 19.5, p < 0.001	X^2 ^= 7.9, p = 0.019	-
45-64	STS	23.3 (22.3 to 24.3)	20.3 (19.3 to 21.3)	20.5 (19.5 to 21.5)
	GLF	20.8 (19.5 to 22.2)	20.8 (19.3 to 22.5)	-
	HSE	21.3 (19.5 to 23.3)	19.2 (18.0 to 20.5)	-
	Heterogeneity	X^2 ^= 0.95, p = 0.008	X^2 ^= 2.76, p = 0.251	-
65+	STS	11.1 (10.2 to 12.1)	9.7 (8.7 to 10.6)	9.4 (8.5 to 10.3)
	GLF	9.5 (8.5 to 10.7)	10.1 (9.0 to 11.4)	-
	HSE	11.2 (9.6 to 13.0)	10.2 (9.2 to 11.3)	-
	Heterogeneity	X^2 ^= 4.6, p = 0.100	X^2 ^= 0.49, p = 0.784	-
SES*				
High SES	STS	17.9 (17.3 to 18.6)	15.3 (14.6 to 16.0)	15.7 (15.1 to 16.4)
	GLF	15.7 (14.8 to 16.7)	16.0 (14.9 to 17.2)	-
	HSE	16.9 (15.5 to 18.4)	17.4 (16.5 to 18.4)	-
	Heterogeneity	X^2 ^= 12.6, p = 0.002	X^2 ^= 11.4, p = 0.0003	-
Low SES	STS	31.9 (31.0 to 32.8)	30.4 (29.4 to 31.4)	29.1 (28.1 to 30.0)
	GLF	27.5 (26.1 to 29.0)	29.3 (27.6 to 31.0)	-
	HSE	29.8 (27.8 to 31.9)	28.0 (26.6 to 29.5)	-
	Heterogeneity	X^2 ^= 26.2, p < 0.001	X^2 ^= 7.3, p = 0.027	-

**†**Data in 2009 is only available for GLF and HSE in published reports and is not appropriately split by age and socio-economic status. Confidence intervals for GLF and HSE in 2009 are estimated based on actual base sample size and confidence intervals in previous years.

*STS = social grade split ABC1 (high SES) & C2DE (low SES), HSE = NSSEC (household) split Professional/Managerial/Intermediate (high SES) & Routine/Manual (low SES).

In 2009 the question to assess prevalence used in GLF and HSE was piloted in the STS. It gave an estimate of 21.5%, the same as from the standard STS question, and similar to the published GLF (21%) and HSE (22%) figures.

There were no differences in the estimates of cigarette consumption between the surveys (Table 5) except among men and 16-24 year olds in 2008, where STS consumption was higher than GLF (X2 = 4.7, p = 0.030) and higher than both GLF (X2 = 3.9, p = 0.042) and HSE (X2 = 6.44, p = 0.048), respectively.

**Table 5:** Mean (standard error) cigarettes per day by sociodemogrpahics for each survey from 2007-2009 where available

		2007	2008	**2009**†
**Total**	STS	13.4 (0.12)	13.4 (0.13)	13.1 (0.12)
**population**	GLF	13.1 (0.19)	13.1 (0.23)	-
	HSE	13.2 (0.27)	13.0 (0.17)	-
	Heterogeneity	X^2 ^= 1.9, p = 0.379	X^2 ^= 3.9, p = 0.145	
**Men**	STS	14.1 (0.17)	14.3 (0.20)	13.8 (0.19)
	GLF	13.9 (0.30)	13.5 (0.31)	-
	HSE	14.0 (0.41)	13.7 (0.24)	13.6 (0.52)
	Heterogeneity	X^2 ^= 0.35, p = 0.839	X^2 ^= 6.2, p = 0.044	X^2 ^= 0.13, p = 0.718
**Women**	STS	12.6 (0.15)	12.6 (0.18)	12.4 (0.16)
	GLF	12.3 (0.23)	12.7 (0.28)	-
	HSE	12.4 (0.30)	12.2 (0.18)	12.6 (0.41)
	Heterogeneity	X^2 ^= 1.3, p = 0.520	X^2 ^= 3.4, p = 0.181	X^2 ^= 0.21, p = 0.650
**16-24**	STS	11.0 (0.20)	12.0 (0.27)	10.8 (0.21)
	GLF	10.6 (0.38)	10.7 (0.60)	-
	HSE	11.4 (0.73)	11.1 (0.35)	-
	Heterogeneity	X^2 ^= 1.3, p = 0.525	X^2 ^= 6.4, p = 0.040	
**25-44**	STS	13.0 (0.18)	13.0 (0.20)	12.4 (0.18)
	GLF	12.6 (0.31)	12.5 (0.34)	
	HSE	12.5 (0.39)	12.4 (0.24)	
	Heterogeneity	X^2 ^= 2.1, p = 0.342	X^2 ^= 4.2, p = 0.125	
**45-64**	STS	15.6 (0.24)	15.5 (0.27)	15.6 (0.25)
	GLF	14.9 (0.31)	15.1 (0.35)	
	HSE	15.1 (0.44)	15.2 (0.29)	
	Heterogeneity	X^2 ^= 3.4, p = 0.180	X^2 ^= 0.99, p = 0.608	
**65+**	STS	13.5 (0.38)	12.9 (0.44)	13.5 (0.45)
	GLF	13.1 (0.58)	12.7 (0.54)	-
	HSE	13.5 (0.65)	13.2 (0.49)	-
	Heterogeneity	X^2 ^= 0.36, p = 0.835	X^2 ^= 0.49, p = 0.784	
High SES*	STS	12.2 (0.17)	12.2 (0.21)	12.1 (0.18)
	GLF	12.2 (0.27)	12.5 (0.33)	-
	HSE	12.6 (0.39)	11.7 (0.23)	-
	Heterogeneity	X^2 ^= 0.93, p = 0.629	X^2 ^= 4.7, p = 0.097	
**Low SES**	STS	14.2 (0.15)	14.2 (0.17)	13.8 (0.16)
	GLF	14.1 (0.27)	13.9 (0.29)	-
	HSE	13.8 (0.38)	14.0 (0.22)	-
	Heterogeneity	X^2 ^= 0.98, p = 0.612	X^2 ^= 1.0, p = 0.602	

*STS = social grade, HSE = NSSEC (household) split Professional/Managerial/Intermediate & Routine/Manual

†Data in 2009 for GLF and HSE is only available from published reports. Only HSE reported mean cigarette consumption, split by gender is available.

No comparison is made between STS follow-up data and other surveys. However, compared with baseline STS smokers and recent ex-smokers who did not provide follow-up data, those responding at three months were significantly older (difference = 4.3 years, t = -15.13, p < 0.001) and were more likely to be female (difference = 7.8%, X2 = 82.1, p < 0.001). There was no difference by social grade (X2 = 1.2, p = 0.281). Those baseline smokers and ex-smokers who provided follow-up data at six months were also significantly older (t = -21.16, p < 0.001) and more likely to be female (X2 = 55.1, p < 0.001) than those who were not successfully followed-up. Again there was no difference by social grade (X2 = 0.04, p = 0.833).

## Discussion

The Smoking Toolkit Study provides monthly nationally representative data on key indicators of smoking behaviour, cessation, and tobacco control initiatives. The STS methodology appears to be robust and reliable, yielding similar results to well-established surveys from the UK on common measures despite some methodological differences.

Prevalence data in 2008 and 2009 were comparable across all surveys. However, in 2007 all three surveys produced significantly different prevalence rates. It is unclear why this might be. A potential explanation relates to the number of policy changes that occurred in 2007, including the banning of smoking in enclosed public places and the change in age of sale of tobacco from age 16 to 18. STS monthly prevalence fluctuated substantially over 2007 [12]. However, STS does not stand out as anomalous in comparison with the other surveys. Prevalence by gender and age were broadly similar across the surveys, although some differences are notable in 2007, probably also reflecting the differences in total population prevalence described above and so again it is unclear as to why this might be. Differences in prevalence by socioeconomic status were observed in both 2007 and 2008. This is not surprising given the different measures used, but despite this a clear and similar pattern in prevalence by SES is apparent in all three surveys with smoking prevalence consistently higher among those in lower SES groups. Levels of cigarette consumption on the other hand were very similar across all surveys and years.

Prevalence data from the GLS and HSE were unavailable for 2010 at the time of writing, highlighting the current lack of up-to-date figures from these surveys. It should also be reiterated that the 2009 data were taken from published survey reports and smoking prevalence for certain sub-groups was therefore not available. Confidence intervals for GLF and HSE in 2009 were estimated based on actual base sample size and confidence intervals from previous years and may therefore be slightly inaccurate. This further draws attention to the lack of available recent data from these surveys. STS results are available shortly after collection and data accessible at request.

As with all studies, there are certain limitations that apply to the STS specifically, and surveys in general. The data presented here are all estimates of smoking prevalence in the population. Although we have shown that the three surveys provide fairly similar estimates, they may all not fully reflect true smoking prevalence. This could be due to inaccuracies in the responses of participants,[29] or perhaps a tendency for smokers to be less likely to agree to participate in such surveys in the first place. Although such nationally representative survey data are considered the 'gold standard' in obtaining prevalence statistics, this caveat should be recognised when citing prevalence estimates. More specific to the STS, data on the methodology and response rates for the follow-up surveys are presented, although it is not appropriate to report prevalence figures as only smokers and recent ex-smokers are surveyed. The response rate for follow-up is low, as is the return of useable saliva samples. However, data are available to establish the representativeness of the follow-up samples on key variables by comparison with those not followed up. Other limitations of the STS include the lack of data on ethnicity at baseline or follow-up. The impact of ethnicity on key parameters cannot therefore be established, although the proportions in each individual ethnic group would not yield a big enough sample to be useful, even with the very large aggregate sample size. Special studies are required to investigate smoking patterns in these groups. Finally, the STS is restricted to data from England, so cannot document the whole of the UK.

The STS has several key strengths, including the ability to examine changes in prevalence and other key performance indicators, such as quit attempts and motivation to quit, on a timely basis and track changes monthly. This permits a more sensitive test of the possible effects of interventions than can be achieved by annual surveys. It also collects sufficient information on characteristics of quit attempts and other relevant variables to provide information on methods of quitting and how these relate to success rates and contextual variables. The large sample size and frequent follow-up allow for these relationships to be accurately estimated and tested prospectively. Indeed, the STS has already produced several important findings, some of which are briefly summarised below.

In terms of policy changes, the STS was able to show that there was a significant temporary increase in smokers attempting to stop by about 300,000 following the introduction of Smokefree legislation [12]. Equally, there is evidence from the STS that the increase in the legal age of sale from 16 to 18 was followed by a decrease in smoking prevalence in this age group, suggesting that these legislative changes can have a real impact on smoking behaviour [9]. Data from the STS also indicate that the general public is much less averse to radical policy changes, such as raising the price of cigarettes to fund tobacco control activities or a total ban on the sale of tobacco products, than is usually assumed [11,15].

Regarding the process of smoking cessation, STS data indicate that enjoyment and stress relief are the most popular reasons given for continued smoking, with men more likely to report enjoyment and women stress relief [10]. While enjoyment of smoking is seen by smokers as a major barrier to stopping,[8] ex-smokers overwhelmingly report being happier than when they were smoking [16]. This provides some reassurance to would-be quitters that their quality of life is likely to improve if they stop [16]. Data from the STS also suggest that there are significant differences in reported triggers for quit attempts as a function of socio-demographic factors. Smokers with higher SES are more likely to report concern about future health whereas those from lower SES are more likely to cite cost and current health problems [17]. However, lower SES smokers are not less likely to try to stop smoking but are less likely to succeed, indicating that structural factors have an important role in aiding smoking cessation [14]. Moreover, failed quit attempts lasting less than a week are quickly forgotten,[6] underlining the need for the frequent assessment of smoking key parameters, with cigarette dependence being the main determinant of the success of quit attempts [8]. There is also evidence that a simple rating of the strength of urges to smoke during a normal smoking day may be a better measure of cigarette dependence than those currently used [7]. Lastly, data from the STS have shed light on the use of support for smoking cessation, finding that half of all quit attempts are aided by some form of pharmacological or behavioural treatment [13]. However, the use of the most effective treatment option, the National Stop Smoking Services, is relatively low[13] and pharmacological support is increasingly employed to aid smoking reduction and temporary abstinence [5].

## Conclusions

In conclusion, the Smoking Toolkit Study provides reliable, up-to-date data on key smoking parameters in England and has already yielded valuable insights into smoking patterns at a national level. Key findings are published on a dedicated website: http://www.smokinginengland.info. Data from the STS have the potential to continue to enhance our understanding of smoking behaviours and provide important contributions to future tobacco control policy and the development of new smoking cessation interventions.

## Competing interests

RW undertakes research and consultancy for the following developers and manufacturers of smoking cessation treatments; Pfizer, J&J, McNeil, GSK, Nabi, Novartis and Sanofi-Aventis. RW also has a share in the patent of a novel nicotine delivery device. AMC has received travel funding, honorariums and consultancy payments from manufacturers of smoking cessation products (Pfizer, J&J, McNeil, GSK, Nabi, Novartis and Sanofi-Aventis). He also receives payment for providing training to smoking cessation specialists; receives royalties from books on smoking cessation and has a share in a patent of a nicotine delivery device. JF, LS, OW, MJJ, JS, and EV have nothing to declare in relation to this paper.

## Authors' contributions

JAF carried out the analyses and drafted the manuscript. LS drafted the manuscript. OW drafted the manuscript. MJJ carried out analyses and critically revised the manuscript. AMc critically revised the manuscript. JS advised on analyses and critically revised the manuscript. EV critically revised the manuscript. RW conceived of the study and critically revised the manuscript. All authors have read and approved the final manuscript.

## Pre-publication history

The pre-publication history for this paper can be accessed here:

http://www.biomedcentral.com/1471-2458/11/479/prepub
